# Supplementary material for: Prevalence and Risk Factors of Musculoskeletal Disorders in Basketball Players: Systematic Review and Meta-Analysis
Source: Healthcare (Basel). 2023 Apr 21;11(8):1190. doi: 10.3390/healthcare11081190 (PMC10138073; doi:10.3390/healthcare11081190)
Supplement: Supplementary file 1 [file healthcare-11-01190-s001.zip › healthcare-2298321-supplementary.pdf]

Supplementary material: Table S1. Database search strategy

**PubMed**	**((musculoskeletal diseases[MeSH Terms])) OR ("musculoskeletal disease"[Title/Abstract])) OR ("musculoskeletal diseases"[Title/Abstract])) OR ("musculoskeletal disorder"[Title/Abstract])) OR ("musculoskeletal disorders"[Title/Abstract])) OR ("musculoskeletal injuries"[Title/Abstract])) OR ("musculoskeletal injury"[Title/Abstract])) OR (posture[MeSH Terms])) OR (posture[Title/Abstract])) OR ("postural evaluation"[Title/Abstract])) OR ("postural changes"[Title/Abstract])) OR (scoliosis[Title/Abstract])) OR (scoliosis[MeSH Terms])) OR (kyphosis[MeSH Terms])) OR (kyphosis[Title/Abstract])) OR (lordosis[Title/Abstract])) OR (lordosis[MeSH Terms])) OR (back pain[MeSH Terms])) OR ("back pain"[Title/Abstract])) OR ("back injuries"[Title/Abstract])) OR (back injuries[MeSH Terms])) OR ("lumbar pain"[Title/Abstract])) OR ("neck pain"[Title/Abstract])) OR (neck pain[MeSH Terms])) OR ("cervical pain"[Title/Abstract])) OR ("spinal pain"[Title/Abstract])) OR ("spine pain"[Title/Abstract])) OR (backache[Title/Abstract])) OR (backaches[Title/Abstract])) OR ("back ache"[Title/Abstract])) OR ("back aches"[Title/Abstract])) OR ("abnormalities in spine"[Title/Abstract])) OR (low back pain[MeSH Terms])) OR ("low back pain"[Title/Abstract])) OR ("cumulative trauma disorders"[Title/Abstract]))) AND (((((((((players[Title/Abstract]) OR (player[Title/Abstract])) OR (sportsman[Title/Abstract])) OR (sportsmen[Title/Abstract])) OR (sportswoman[Title/Abstract])) OR (sportswomen[Title/Abstract])) OR (athletes[MeSH Terms])) OR (athletes[Title/Abstract])) OR (athlete[Title/Abstract])))))) AND (((((basketball[MeSH Terms]) OR (basketball[Title/Abstract])) OR (basket[Title/Abstract]))) OR (sports[Title/Abstract])) OR (sport[Title/Abstract])**
Scopus	(TILE-ABS (basket OR basketball OR sports OR sport)) AND (TITLE-ABS (players OR player OR sportsan OR athletes OR athlete OR sportsmen OR sportswoman OR sportswomen)) AND (TITLE-ABS ("musculoskeletal disorder" OR "musculoskeletal disorders" OR "musculoskeletal disease" OR "musculoskeletal diseases" OR "musculoskeletal injuries" OR "musculoskeletal injury" OR posture OR "postural evaluation" OR "postural chances" OR scoliosis OR kyphosis OR lordosis OR "back pain" OR "low back pain" OR "back injuries" OR "lumbar pain" OR "neck pain" OR "cervical pain" OR "spinal pain" OR "spine pain" OR backache OR backaches OR "back ache" OR "back aches" OR "abnormalities in spine" OR "cumulative trauma disorders"))
Embase	(posture:ab,ti OR 'postural evaluation':ab,ti OR 'postural changes':ab,ti OR scoliosis:ab,ti OR kyphosis:ab,ti OR lordosis:ab,ti OR 'musculoskeletal disorder':ab,ti OR 'musculoskeletal disorders':ab,ti OR 'musculoskeletal disease':ab,ti OR 'musculoskeletal diseases':ab,ti OR ‘musculoskeletal injuries’:ab,ti OR ‘musculoskeletal injury’:ab,ti OR 'back pain':ab,ti OR 'low back pain':ab,ti OR 'back injuries':ab,ti OR 'lumbar pain':ab,ti OR 'neck pain':ab,ti OR 'spinal pain':ab,ti OR 'cervical pain':ab,ti OR 'spine pain':ab,ti OR backache:ab,ti OR backaches:ab,ti OR 'back ache':ab,ti OR 'back aches':ab,ti OR 'cumulative trauma disorders':ab,ti OR 'abnormalities in spine':ab,ti) AND (players:ab,ti OR player:ab,ti OR sportsman:ab,ti OR athletes:ab,ti OR sportsmen:ab,ti OR sportswoman:ab,ti OR athlete:ab,ti) AND (basket:ab,ti OR basketball:ab,ti OR sports:ab,ti OR sport:ab,ti)
